# Specifying the Probability Characteristics of Funnel Plot Control Limits: An Investigation of Three Approaches

**DOI:** 10.1371/journal.pone.0045723

**Published:** 2012-09-20

**Authors:** Bradley N. Manktelow, Sarah E. Seaton

**Affiliations:** Department of Health Sciences, University of Leicester, Leicester, United Kingdom; Queen’s University Belfast, United Kingdom

## Abstract

**Background:**

Emphasis is increasingly being placed on the monitoring and comparison of clinical outcomes between healthcare providers. Funnel plots have become a standard graphical methodology to identify outliers and comprise plotting an outcome summary statistic from each provider against a specified ‘target’ together with upper and lower control limits. With discrete probability distributions it is not possible to specify the exact probability that an observation from an ‘in-control’ provider will fall outside the control limits. However, general probability characteristics can be set and specified using interpolation methods. Guidelines recommend that providers falling outside such control limits should be investigated, potentially with significant consequences, so it is important that the properties of the limits are understood.

**Methods:**

Control limits for funnel plots for the Standardised Mortality Ratio (SMR) based on the Poisson distribution were calculated using three proposed interpolation methods and the probability calculated of an ‘in-control’ provider falling outside of the limits. Examples using published data were shown to demonstrate the potential differences in the identification of outliers.

**Results:**

The first interpolation method ensured that the probability of an observation of an ‘in control’ provider falling outside either limit was always less than a specified nominal probability (*p*). The second method resulted in such an observation falling outside either limit with a probability that could be either greater or less than *p*, depending on the expected number of events. The third method led to a probability that was always greater than, or equal to, *p*.

**Conclusion:**

The use of different interpolation methods can lead to differences in the identification of outliers. This is particularly important when the expected number of events is small. We recommend that users of these methods be aware of the differences, and specify which interpolation method is to be used prior to any analysis.

## Introduction

In recent years there has been increased focus and emphasis placed on the comparison of clinical outcomes between healthcare providers: for example the United Kingdom Department of Health has promised a “*relentless focus on delivering the outcomes that matter most to people*” [1]. Methods taken from Statistical Process Control (SPC) have gained popularity in healthcare and funnel plots have become a standard graphical technique for reporting and comparing clinical outcomes [2]–[5]. Such funnel plots comprise the plotting of an outcomes summary statistic from each individual provider against a specified ‘target’ (in the case of Standardised Mortality Ratios, this is the value one, i.e. the observed number of events equals that expected) together with upper and lower control limits.

The use of funnel plots has been recommended in the UK by groups including the National Clinical Audit Advisory Group [6] and the Association of Public Health Observatories [7]. These organisations recommend that any provider that falls outside the control limits, in particular the upper limit, should be viewed as an outlier, and investigations to discover the reason for this should be undertaken [6], [7]. This decision of whether to investigate or not, can be made even based on a small number of events and identification of a provider can have important consequences for all involved. It is crucial, therefore, that funnel plots are produced and interpreted correctly.

The control limits are constructed with the aim that an observation from a provider with an underlying performance equal to the ‘target’ (i.e. ‘in control’ or performing with the variation expected) will fall above, or below, the control limits with a specific, known probability. However, when the outcome follows a discrete probability distribution it is not possible to specify the exact probability with which an observation from an ‘in-control’ provider will fall outside of the limits [8]. For example, for a sample from a population following a Poisson distribution with a mean of 10 the probability of observing more than 16 events is 0.027 whereas the probability of observing more than 17 events is 0.014. It is not possible to specify a set of outcomes for which the probability is *exactly* 0.025.

Clinical outcomes are very often discrete counts (e.g. number of deaths, infections, post-operative complications) and these are usually compared through the use of the Standardised Mortality Ratio (SMR). The SMR is defined as the ratio of the observed number of events to the number expected using the outcomes of a reference population and it is usually assumed that the observed number of events is an observation from a Poisson distribution [9].

Given that it is not possible to stipulate the exact probability that an observation from an ‘in-control’ provider will fall outside the control limits, because of the discrete nature of the outcome, there are several ways in which the probability characteristics of the control limits for the SMR can be specified. It is often required that the probability of an observation from an ‘in control’ provider falling outside either the upper or the lower control limit is always less than the specified nominal probability: for example, the probability of falling above the upper limit, or below the lower limit, of 95% control limits is always less than 0.025. Another option is to construct the limits so that the probability of an observation of falling outside of the limits is always greater than or equal to the nominal probability. A further approach is some combination of these to obtain limits that produce a probability that is ‘closest’ to the nominal probability.

Interpolation methods are often used to improve the appearance of the control limits on funnel plots by producing smooth, rather than jagged, control limits and also to ensure that no observed point can fall ambiguously directly on the limit [2], [10]–[12]. However, the choice of interpolation method can also be used to obtain the desired probability characteristics from the control limits. It is important, therefore, that the probability characteristics of interpolated control limits on funnel plots are understood in order for them to have the correct interpretation.

In this paper we describe three different interpolation methods and investigate their probability characteristics and interpretation.

## Methods

The Standardised Mortality Ratio (SMR) is defined as the ratio of the observed number of events (*O*) to the expected number of events (*E*): i.e. SMR = *O*/*E*, where *O* is an integer value.

It is assumed that a 100(1–2*p*)% control interval is required. Ideally with such an interval, the probability that an observation from an ‘in-control’ healthcare provider will fall within the control limits is 1–2*p*. Hence, the probability that the observation will fall above the upper control limit is *p* (the ‘nominal probability’), and the probability it will fall below the lower control limit is also *p*. For outcomes that are observations from a discrete probability distribution it is not possible to draw limits which fulfil these criteria exactly and three approximation methods are described below.

### Method 1: Probability SMR from an ‘in-control’ Provider will Fall Outside each Limit is <*p*

The lower control limit *L* can be defined as *o_L_*/*E*, where *o_L_* is the smallest integer such that P(*O* ≤ *o_L_*) >*p*. Similarly, the upper control limit *U* can be defined as *o_U_*/*E*, where *o_U_* is the largest integer such that P(*O* ≥ *o_U_*) >*p* [2].

Since *O* can only take integer values (as it represents an observed number of events) it is not possible for an observed value of the SMR to take a value that is greater than *o_U_/E* but less than (*o_U_*+1)*/E*. Hence, the upper limit *U* can, in fact, take any value in the range *o_U_/E* ≤ *U* < (*o_U_*+1)*/E* and still satisfy the requirement of being the maximum value for which an observed SMR from an ‘in control’ provider will fall above the limit with a probability of less than the nominal probability *p*. Similarly, the lower interpolated limit will lie between *o_L_/E* and *(o_L_−1)/E.*

Method 1 comprises plotting the interpolated upper limit at a weighted average between *o_U_/E* and (*o_U_*+1)/E (Table 1) and the lower interpolated limit will lie between *o_L_/E* and *(o_L_−1)/E* [2]. Therefore, the upper limit will be constrained to lie between *o_U_* and (*o_U_*+1) and the lower limit between *o_L_* and (*o_L_−*1).

**Table 1:** Formulae for three methods for calculating values of the interpolation upper and lower control limits for the funnel plots based on the Poisson distribution.

	Upper control limit	Lower control limit
**Method 1**	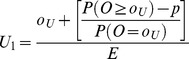	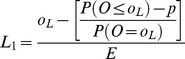
**Method 2**	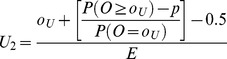	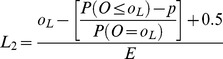
**Method 3**	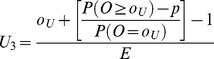	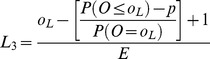

Where *o_U_* is the largest integer such that P(*O* ≥ *o_U_*) ≥*p* and *o_L_* is the smallest integer such that P(*O* ≤ *o_L_*) ≥*p*.

### Method 2: Probability SMR from an ‘in-control’ Provider will Fall Outside each Limit is Closest to *p*

An alternative interpolation method (Method 2) comprises drawing the control limits ‘closest’ to the nominal probability *p* [10] (Table 1). The interpolated value of the upper control limit is constrained to take a value from (*o_U_−*0.5)/*E* up to, but not including, (*o_U_*+0.5)/*E*. Similarly, the lower control limit will lie in the region from (*o_L_*+0.5)*/E* down to, but not including, (*o_L_−*0.5)*/E*. Since the values of the interpolated upper control limit can take values both less than and greater than *o_U_*/*E*, the probability that the observed SMR from an ‘in control’ provider will fall above the upper limit, or below the lower limit, can be greater than or less than *p* depending on the expected number of events. This method ensures that on average the probability of ‘in-control’ providers falling outside each limit is *p*. This is similar to the use of mid-*P* confidence intervals [13].

### Method 3: Probability SMR from an ‘in-control’ Provider will Fall Outside each Limit is ≥*p*

The third proposed method is produced in a similar way to Method 1. However, under this method the interpolated value of the upper control limit will lie from (*o_U_−*1)*/E* up to, but not including, *o_U_/E*. Similarly the lower limit is specified to lie from (*o_L_*+1)*/E* down to, but not including, *o_L_/E*. Therefore, in this case the probability of an observation falling above the upper limit or below the lower limit is always greater than or equal to the nominal probability *p*. Method 3 comprises plotting the upper control limit at a point that is a weighted average between than *o_U_* and (*o_U_−*1) and the lower limit between *o_L_* and (*o_L_+*1) (Table 1).

### Statistical Analysis

The three interpolation methods were initially compared by plotting the true probability of an observation from an ‘in-control’ provider falling outside the upper and lower 95% control limits against the expected number of events (*E*) for each of the methods up to *E*≤30. The median probability was calculated for *E*≤10 and compared to 250<*E*≤500.

These probabilities were calculated directly using the cumulative probability distribution of the appropriate Poisson distribution.

### Data for Examples

To illustrate the differences between these interpolation methods in identifying outliers, two examples using published data were investigated.

In the first example data were used from the review of paediatric cardiac services at the Oxford John Radcliffe Hospital NHS Trust [14]. Table 5 in Appendix A of the report shows the data for 30-day mortality following paediatric cardiac surgery by different surgical procedures at the Oxford John Radcliffe Hospital NHS Trust from 2000–2008.

In the second example the data were taken from the 2010 report of The Neonatal Survey (TNS), a survey of neonatal care in the East Midlands and Yorkshire regions of the UK [15]. The data presented here are for death before discharge by Primary Care Trust (PCT) of very preterm babies (20 to 32 weeks gestational age at birth) born from 2008 to 2010 and admitted to neonatal care (Table 28 in the report).

For the examples, the reported SMRs were plotted on the funnel plot showing 95% control limits calculated using the three interpolation methods.

SAS v 9.2 was used for all analyses and to produce the Figures.

## Results

**Figure 1:**
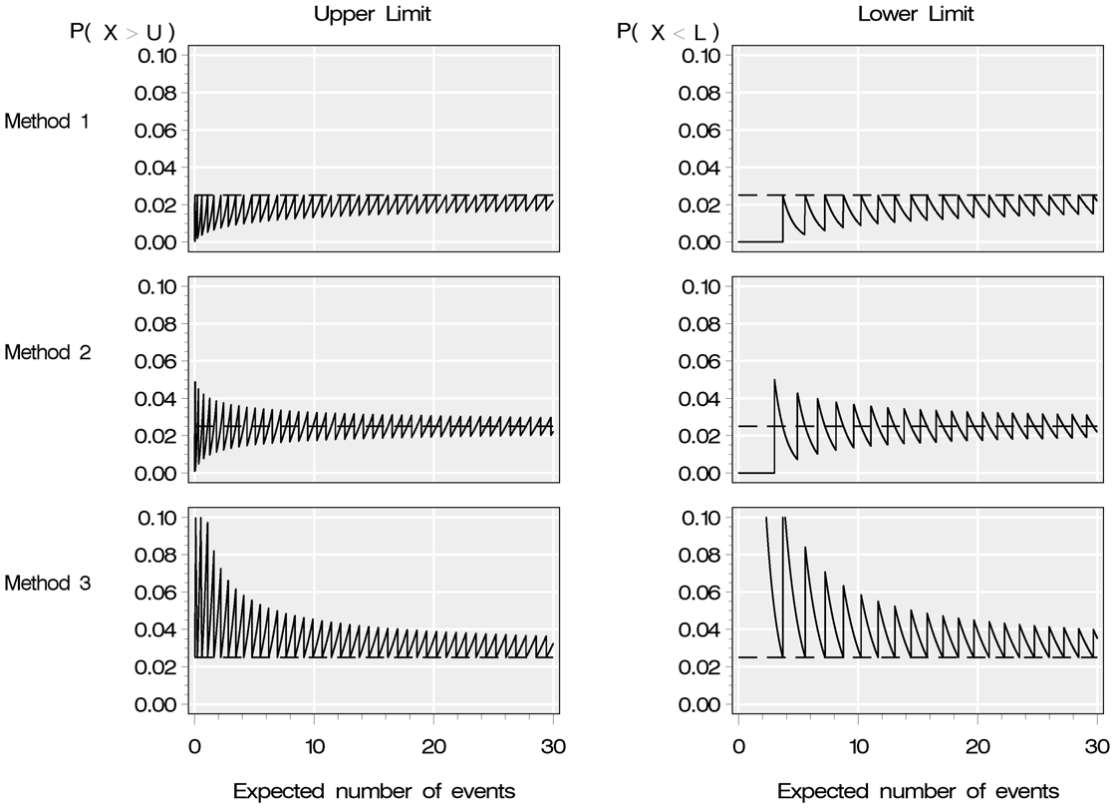
Probability of falling outside control limits. Probability of identification of falling above the upper limit or below the lower limit for three different interpolation methods.

The probabilities of an observation from an ‘in-control’ provider falling outside of the upper and the lower 95% control limits were plotted against the expected number of events (*E*) for each of the interpolation methods for *E*≤30 (Figure 1). The dashed line on each plot represents the nominal probability *p*: 0.025 in the case of 95% intervals.

For Method 1 (first row of Figure 1) when *E*≤10 the median probability of an observation from an ‘in-control’ provider falling above the upper limit was 0.0160 (range 0.0003 to 0.0250) and the median probability of falling below the lower limit was 0.0084 (range 0.0000 to 0.0250). For 250<*E*≤500 the median probability of falling above the upper limit was 0.0236 (range: 0.0217 to 0.0250) and below the lower limit was 0.0235 (range: 0.0214 to 0.0250).

As expected, with Method 2 (second row of Figure 1) the probability of an ‘in-control’ provider falling outside each limit is both greater and less the nominal probability (*p*) depending on the value of *E*. The median probability of falling above the upper limit was 0.0228 (range: 0.0010 to 0.0488) and below the lower limit was 0.0163 (range: 0.0000 to 0.0500) for *E*≤10. When the *E* was larger, the probability tended towards the nominal probability and the median probability of falling above the upper limit when *E* was 250<*E*≤500 was 0.0250 (range: 0.0231 to 0.0269) and the median probability of falling below the lower limit was 0.0250 (range: 0.0231 to 0.0269).

The median probability of being identified as falling above the upper limit with Method 3 (third row of Figure 1) for *E*≤10 was 0.0383 (range: 0.0250 to 0.2149) and below the lower limit was 0.0554 (range: 0.0250 to 0.9990). When the expected number of events was larger (250<*E*≤500) the median probability of falling above the upper limit was 0.0265 (range: 0.0250 to 0.0288) and the median probability of falling below the lower limit was 0.0266 (range: 0.0250 to 0.0291). For the lower limit of this method, when *E*<2.3 the probability of falling below the lower limit is greater than 0.1, and these values are missing from Figure 1 as they fell outside the range used for the vertical axis on the figure.

### Examples of Implementation

Oxford John Radcliffe Hospital NHS Trust data [14].

**Figure 2:**
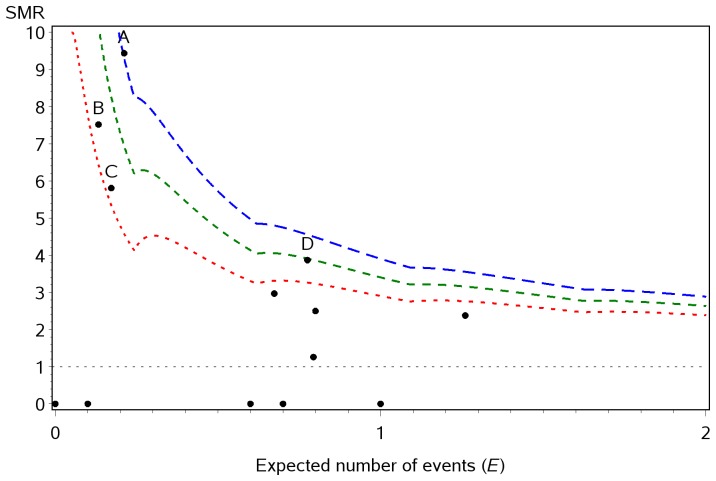
Example using data from the Oxford John Radcliffe Hospital NHS Trust. Mortality following paediatric cardiac surgery at the Oxford John Radcliffe Hospital NHS Trust 2000–2008 by surgical procedure group, with 95% control limits calculated using three interpolation methods: Method 1 (blue); Method 2 (green) and Method 3 (red).

The data from the Oxford John Radcliffe Hospital NHS Trust are shown in Figure 2, where the expected number of events (*E*) was less than 2 and the limits constructed using the three different methods are shown. It can be seen that although interpolation methods have been used and the control limits ‘smoothed’ these do not necessarily produce monotonic functions. Only one point fell above the upper control limit obtained using Methods 1 and 2: point A. However, three further points (B, C & D) fell above the limits calculated using Method 3 and would have been identified as potential outliers using the limits created by this interpolation. As the difference between the methods represents, at most, one observed event, points B, C and D would be identified as potential outliers by all of the methods if the number of observed deaths for each of these surgical procedures was increased by one.

The Neonatal Survey (TNS) [15].

**Figure 3:**
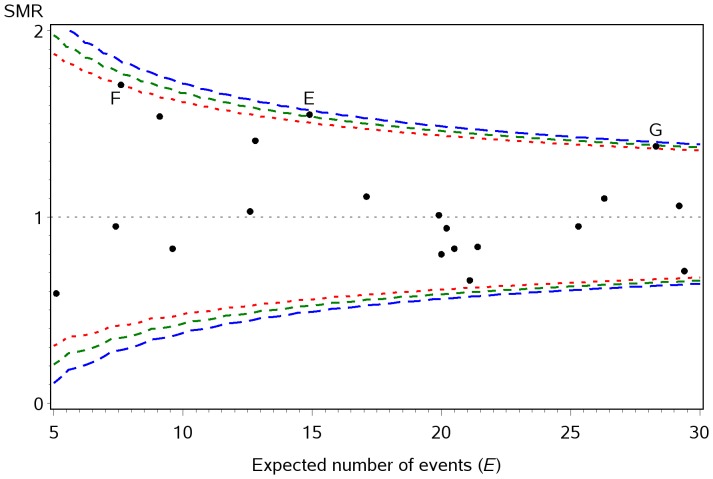
Example using data from The Neonatal Survey. Mortality before discharge from neonatal care for babies born at 20 to 32 weeks gestational age 2008–2010 by PCTs in the East Midlands and Yorkshire, with 95% control limits calculated using three interpolation methods: Method 1 (blue); Method 2 (green) and Method 3 (red).

Using the data from The Neonatal Survey (TNS), no points fell above the upper control limits calculated using Method 1: shown in Figure 3 for values of *E* from 5 to 30. However, point E falls above the upper limit obtained from Method 2 and would be identified as a potential outlier using this interpolation method. Two further points (F & G) lie above the limits obtained using Method 3.

Therefore, use of Method 1 would result in no PCTs being identified as potential outliers, Method 2 would result in one PCT being identified and Method 3 would result in three PCTs being identified.

## Discussion

Funnel plot limits are increasingly used as a method for displaying outcomes from healthcare providers. Providers which are identified as falling outside the funnel plot control limits are viewed as outliers and usually then subject to some form of investigation [6], irrespective of whether they are identified correctly or not. This can have important consequences for these healthcare providers. SPC methods, including funnel plots, are being used in inquiries into healthcare providers: for example, in the UK they were used in both the review of paediatric surgery at Oxford Radcliffe Hospitals NHS Trust [14] and the Healthcare Commission’s Investigation into Mid Staffordshire NHS Foundation Trust [16].

It has been shown that when the outcome statistic is based on count data (in this case the SMR) it is not possible to specify the exact probability of an observation from an ‘in-control’ provider falling outside of the control limits [8]. In the light of this, different approaches can be taken to specify the probability characteristics of the control limits and three possibilities have been described in this paper. Although these interpolation methods have already been seen in medical research [14], [17], the choice of which approach should be used in practice is not always obvious. Under Method 1 the probability of an observed SMR from an ‘in control’ provider falling above the upper control limit (and similarly below the lower limit) is always less than the nominal probability *p*. This probability can sometimes be substantially less than *p* especially when the expected number of events *E* is small. On the other hand, using Method 3 the probability of such an observation falling outside each limit is always greater than, or equal to, *p*. For small values of *E* the actual probability can be much larger than *p*. Compared to Method 3, the use of Method 1 will reduce the probability of providers being falsely identified as outliers by chance alone (i.e. increased specificity) but will increase the probability of not identifying providers with truly divergent rates of outcome (i.e. reduced sensitivity).

Method 2 offers a compromise as the probability of an observed SMR from an ‘in control’ provider lying outside each of the control limits is ‘on average’ equal to *p*. This probability is either greater than or less than *p* depending on the value of *E*. However, it is not obvious whether the probability is greater or less than *p* at any particular value of *E*, in other words for any particular healthcare provider. Although as the true probability is not constant across all values of *E* for any of the methods (and can never be so) this characteristic could perhaps be viewed as only a minor disadvantage of Method 2. However, Methods 1 and 3 do at least allow us to know that the probability will always be less than (Method 1), or greater than (Method 3), the nominal probability.

This issue is not unique to funnel plot control limits but also arises when estimating confidence intervals for any discrete probability distribution [18], [19]. To paraphrase Agresti and Coull, in forming 95% control limits for a funnel plot, is it better to use an approach that guarantees that the actual probability of falling above the upper limit is *not more than* 0.025 yet typically achieves probabilities of 0.016 (when *E*≤10), or an approach giving narrower intervals for which the actual probability could be greater than 0.025 but is quite close to 0.025? The answer to that is likely to depend on the risks and benefits of providers being identified, falsely or otherwise, as outlier. If, for example, the outcome being compared is paediatric death, a conservative approach increasing the probability of identifying healthcare providers with truly divergent rates of poor outcome may be wanted and Method 3 the most appropriate choice with a risk that some providers will be unnecessarily investigated. Conversely, if the risks and costs of unnecessary investigation of healthcare providers falsely identified outweigh the risk of missing some providers with divergent rates of outcome, for example minor infection, Method 1 may be most appropriate choice. However, in the absence of special circumstances perhaps Method 2 should be the default method as it has the characteristic that the probability that an observation from an ‘in-control’ provider will fall outside either control limit with a probability that overall is closest to the nominal probability: although for any particular provider this may be far from the nominal probability.

In reality, the differences seen between the interpolation methods were particularly apparent and potentially important when the expected number of events was small. Indeed the difference in the limits from the three methods is, at most, only 1 observed event. Such a difference is unlikely to be important when the number of events is large. However, this can be an important difference when small datasets are used or the outcome is rare. Funnel plot control limits are used, and are increasingly likely to be used, to try to identify potentially poorly performing healthcare providers when the number of events is small. While the reporting of hospital-wide outcome statistics such as the Summary Hospital–level Mortality Indicator (SHMI) [20] and Hospital Standardised Mortality Ratio (HSMR) [21] have been advocated, such a general approach can be difficult to interpret [22], [23]. The use of more focused data from clinical specialities has been recommended [24]–[26] but the more frequent use of these small datasets increases the need to fully understand the characteristics of the statistical methods used in this context.

Our examples, using data from the Oxford John Radcliffe Hospital NHS Trust and The Neonatal Survey, have shown that the choice of interpolation method can result in the labelling of a provider as a potential outlier using one method whilst not by another, leading to real implications for that provider. Funnel plots are recommended to be used to identify providers for further investigation with potentially outlying performance [6] and it is clear that the use of different interpolation methods could lead to different conclusions. If the probability properties are not understood correctly this could cause unnecessary investigations for hospitals and other care providers, leading to wasted money, time and reputation or causing providers with truly outlying outcomes to not be identified. It is important that any interpolation method used is specified a priori as the different probability properties of the methods allows the potential for ‘gaming’; i.e. choosing the method that produces the answer wanted.

There are further methods for choosing the interpolated value for the control limits. However other potential methods were not investigated in this paper because they offered no advantage over the three methods included here. For example the “midpoint convention”, i.e. (*x_U_*+0.5)/E [12], has the same probability characteristics as Method 1. Also, in this paper it has been assumed that it is desirable that the limits be symmetrical: that is, both the upper and lower limits have the same probability characteristics. While it is difficult to imagine why this would not be required, it is possible to use different interpolation methods on the upper and lower limits to create asymmetrical intervals.

Whilst this paper has focused on the SMR based on the Poisson distribution, the interpolation methods described here have also been advocated for outcomes based on other discrete probability distributions (e.g. binomial, negative binomial). The arguments and conclusions set out in this paper will hold whichever discrete distribution is assumed.

### Conclusions

Interpolation methods can be used to improve the aesthetics and interpretability of control limits on funnel plots as well as set their probability characteristics. It has been shown in this paper that the different interpolation methods presented alter the probability of a health care provider being identified as an outlier. Care should be taken to understand the properties of the limits drawn before using them to identify outliers. All methods here potentially have a use depending on the clinical question of interest but the choice of method should be undertaken prior to analysis.
